# Analysis of health-related quality of life and costs based on a randomised clinical trial of escitalopram for relapse prevention in patients with generalised social anxiety disorder

**DOI:** 10.1111/j.1742-1241.2008.01879.x

**Published:** 2008-11

**Authors:** C François, S A Montgomery, N Despiegel, S Aballéa, J Roïz, P Auquier

**Affiliations:** 1 Global Outcomes and HTA Division, Lundbeck SASParis, France; 2 Imperial CollegeLondon, UK; 3 i3 InnovusUxbridge, UK; 4 Département de Santé Publique, Faculté de MédecineMarseille, France

## Abstract

**Background:**

Social anxiety disorder (SAD) is associated with substantial reduction in health-related quality of life (HRQoL). Escitalopram has proven efficacy in the short-term treatment of SAD and prevention of relapse.

**Objectives:**

To determine whether the clinical effects of treatment translated into HRQoL benefits and to investigate costs of SAD treatment.

**Methods:**

Data on HRQoL and resource utilisation were collected in a previously published clinical trial of escitalopram in relapse prevention. Among 517 patients, 371 responded to 12 weeks of open-label treatment with escitalopram and were randomised to escitalopram or placebo for 24 weeks. HRQoL was assessed using the short form (SF)-36 instrument and SF-6D utilities (preference-based index scores for overall HRQoL) were calculated. Costs were calculated for responders over the acute phase and for non-relapsed patients over the continuation phase, applying UK unit costs.

**Results:**

Health-related quality of life was significantly improved after the acute phase when compared with baseline. The SF-6D utility increased by 0.047 in responders (p < 0.0001) and 0.021 in non-responders (p = 0.0005). Healthcare costs were non-significantly lower in acute phase than during prestudy phase (p = 0.0587 from NHS perspective), as were productivity costs (p = 0.1440). HRQoL at last visit was lower in relapsed than non-relapsed patients. The difference in utility was −0.026 (p = 0.0007). Healthcare and productivity costs were non-significantly lower in the escitalopram group than in the placebo group.

**Conclusions:**

Both effective acute treatment of SAD and prevention of relapse with escitalopram are associated with significant HRQoL benefits. Despite some limitations, the cost analysis suggests that savings in physician-visits and inpatient care may offset drug acquisition costs.

**What’s known**

- Escitalopram is effective in the treatment of patients with generalised social anxiety disorder (SAD) and the prevention of relapse.
- Health-related quality of life is substantially impaired in patients with SAD.

**What’s new**

- Acute treatment of SAD and prevention of relapse with escitalopram have positive effects on HRQOL.
- Drug acquisition costs associated with escitalopram were offset by savings in physician-visits and inpatient care in the studied sample.

## Introduction

Social phobia is a commonly occurring anxiety disorder often associated with serious role impairment (1). Social anxiety disorder (SAD) can be classified into two subtypes: ‘discrete’ or ‘specific’ and ‘generalised’.

Generalised SAD, also known as generalised social phobia, is defined as a persistent fear of most social or performance situations in which one is exposed to unfamiliar people or to possible scrutiny by others (2). In the ‘discrete’ or ‘specific’ subtype, the patients usually have public-speaking fears only. Generalised social phobia is more severe and disabling than other social phobias. The annual prevalence of SAD is 7–8% and lifetime prevalence is 12–14% (1,3). Generalised SAD represents two-thirds of social phobias (4). Data from the United States (2001–2002) showed that the mean age at onset of SAD was 15.1 years, with a mean duration of 16.3 years (5). Furthermore, individuals were at an increased risk if they were Native American, young or of low income (5).

Individuals with SAD have a high risk of developing additional anxiety and mood disorders, including suicidal behaviour (6). Additionally, SAD has an adverse impact on other comorbid mental conditions such as bipolar disorder, eating disorders, and personality disorders (3). Independent of these comorbidities, generalised SAD has a significant detrimental effect on health-related quality of life (HRQoL) (7). In addition to its burden on individuals, SAD places a substantial burden on health and social services (8). A study among members of a Health Maintenance Organisation based in the USA found that the average number of outpatient visits per year was higher by 2.5 in patients with generalised SAD and no comorbid psychopathology, compared with those without psychiatric diagnosis (9). Furthermore, subjects with generalised SAD missed a greater percentage of work time than those with no psychiatric diagnosis (2.83% vs. 1.82%).

Established treatments for SAD include cognitive behaviour therapy and selective serotonin reuptake inhibitors (SSRIs). A number of SSRIs, including paroxetine, sertraline and fluvoxamine, have been found to be effective in the treatment of generalised SAD, based on randomised, placebo-controlled, clinical trials (10–14). Furthermore, randomised clinical trials in maintenance treatment over 24 weeks showed that paroxetine (SAD) or sertraline (generalised SAD) was associated with a significant reduction in risk of relapse, compared with placebo (15,16). In addition, escitalopram (Cipralex® Product Monograph, H. Lundbeck AS, Copenhagen, Denmark, 2007), an SSRI with efficacy comparable to paroxetine and more favourable tolerability than paroxetine, is indicated for SAD (17,18).

Montgomery et al. (19) reported the results of a multinational randomised, placebo-controlled trial of escitalopram for the prevention of relapse in generalised SAD. HRQoL and resource utilisation data were collected in association with this trial. Based on the collected data, a secondary analysis was performed to investigate whether the clinical effect of treatment was associated with HRQoL benefits and resource savings. Our overall aim was to fill the gap in the quantitative literature concerning the impact of relapse and response to treatment on HRQoL and costs in patients with generalised SAD. More specifically, the analysis had three objectives: (i) to assess the extent to which HRQoL and costs were influenced by response to acute treatment, (ii) to assess the impact of relapse on HRQoL and (iii) to assess the impact of long-term treatment with escitalopram on HRQoL and costs.

## Material and methods

### Study design and previous findings

The design of the clinical trial on which this analysis is based was described by Montgomery et al. (19). A total of 571 patients with a primary diagnosis of generalised SAD (per Diagnostic and Statistical Manual 4th edition criteria) and Liebowitz Social Anxiety Score (LSAS) ≥ 70 received 12 weeks of open-label treatment with escitalopram. The initial dose of 10 mg/day could be increased to 20 mg/day if clinically indicated. Of these patients, 372 responded to open-label treatment and 371 were randomised in a 1 : 1 ratio, using a computer-generated block-randomisation list, to receive double-blind treatment with escitalopram (*n* = 190) at a fixed dose of 10 or 20 mg, or placebo (*n* = 181). Escitalopram and placebo tablets were identical in smell and appearance, and packed identically. Treatment was continued for 24 weeks unless the patient relapsed or was withdrawn for other reasons. Response was defined as a Clinical Global Impression-Improvement (CGI-I) score of one or two (20). Relapse was defined as either an increase in LSAS total score of ≥ 10 points or withdrawal of the patient from the study because of lack of efficacy as judged by the investigator. The study was conducted across 76 centres in nine European countries, Canada and South Africa from January 2001 to June 2002. Patients from all countries were included in this analysis.

This study showed that the probability of relapse was 2.8 times lower in patients randomised to escitalopram (22% vs. 50% on placebo, p < 0.001). It was concluded that escitalopram is effective and well tolerated for long-term treatment of generalised SAD.

### HRQoL assessments

Health-related quality of life was assessed using the Medical Outcome Study Short Form (SF-36) (21). This is a generic instrument providing eight domain scores: Physical Functioning (PF), Role limitations because of Physical problems (RP), Bodily Pain (BP), General perception of Health (GH), Social Functioning (SF), Role limitations because of Emotional problems (RE), Mental Health (MH) and Vitality (VT). Item scores related to each domain are coded, summed and scaled from zero (worst possible health state) to 100 (best possible health state). A 10-point difference on any domain score is generally accepted as clinically relevant (22).

Patients completed the SF-36 questionnaire at initiation of the acute phase (baseline), the end of the acute phase, and at 12 and 24 weeks after randomisation. Patients who did not complete the study attended an early discontinuation visit, at which the SF-36 was administered.

### Health state utilities

Health state utilities are preference-based index measures of well-being that can be used to estimate quality-adjusted life-years (QALYs) for use in economic evaluations (23). Conventionally, a utility of one represents perfect health and a utility of zero represents a health state considered equivalent to death. QALYs are health outcome measures that combine length and quality of life and are calculated by multiplying the utility given to a health state by the time spent in that state.

Brazier and colleagues (24,25) derived a six-dimensional health state classification, called the SF-6D, from the SF-36 for which a preference-based scoring system has been developed. This system was used to derive health state utilities, i.e. SF-6D scores, from the SF-36 questionnaire.

### Resource utilisation

A resource utilisation questionnaire, specifically developed for the purpose of this study, was administered at 12-week intervals (i.e. at the same visits as SF-36). In this questionnaire, SAD-related resources used over the past 12 weeks were recorded, including physician consultations (excluding consultations carried out for the study), visits to other healthcare professionals and social workers, and hospitalisations. In addition, patients were asked for details of sick leave days related to SAD occurring during the study or the 12 weeks preceding baseline. Resources used after relapse or withdrawals from the study were not recorded. Cost estimates also included acquisition of escitalopram. Counts of returned tablets were recorded at all visits. These data were used to calculate the cost of escitalopram during the acute phase and over the two trimesters (12-week periods) of the continuation phase.

### Estimation of costs

Healthcare resources were valued using unit costs prevailing during 2006 in the UK, from the perspective of the NHS and Personal Social Services (abbreviated as the NHS perspective below), and from the perspective of society. Costs associated with non-conventional medicine, such as consultations with acupuncture specialists or chiropractors, were accounted for from the societal perspective but not from the NHS perspective. Table 1 shows unit costs for key resource items. It was not necessary to discount costs to present value as the timeframe of the study was < 1 year.

**Table 1:** Unit costs (GBP £2006) for key resource items

Resource	Unit cost	Reference
**Escitalopram**		
28 Tablets, 10 mg	£14.91	BNF (35)
28 Tablets, 20 mg	£25.20	
GP visit	£25.00	PSSRU (36)
Psychiatrist consultation (20 min)	£82.00	PSSRU (36)
Psychologist consultation (60 min)	£66.00	PSSRU (36)
Nurse visit	£23.50	PSSRU (36)
Social worker visit (30 min)	£60.00	PSSRU (36)
**Hospitalisation**		
Psychiatric ward (per day)	£294.00	PSSRU (36)
Other ward (per day)	£421.00	National Reference Costs (37)

GP, General Practitioner.

Productivity costs were reported separately from other costs. The cost of a lost work day was based on mean gross UK daily earnings in 2006. A value of £107.46 per sick leave day was applied (26).

As resources used after discontinuation of the study treatment were not systematically collected, cost estimates for each study trimester are based on patients who completed the trimester. Therefore, this analysis only reports costs in responders over the acute phase and in non-relapsed patients over the continuation phase.

### Statistical analyses

Descriptive statistical analyses were performed. Mean and standard deviations were calculated for all HRQoL scores and costs by trimester (12-week period). HRQoL scores at the end of acute phase or discontinuation were compared between responders and non-responders using *t*-tests. Similarly, HRQoL scores were compared between patients who subsequently relapsed and other patients who continued to receive long-term treatment. Data from questionnaires completed at time of relapse were used for patients who relapsed, and data collected at the end of the continuation phase or at withdrawal were used for the remaining patients. Analysis of covariance was used to compare HRQoL scores at the end of the continuation phase, at relapse or upon withdrawal between the two treatment groups, adjusting for scores at randomisation. This adjustment was necessary as HRQoL at randomisation might differ between patients who subsequently relapsed and those who did not, eventhough all were in remission.

Paired *t*-tests were performed to compare costs over the 12 weeks preceding baseline with costs over the acute phase. In addition, cost measurements were compared between the escitalopram and placebo patients for each trimester of the continuation phase using *t*-tests. Healthcare costs were estimated with and without the inclusion of hospitalisations as a small number of costly hospitalisations introduced substantial variability around overall cost estimates.

A level of significance of 5% was used in interpreting results of statistical tests. The hypothesis of equality in quality of life between the two groups of patients or between two visits was rejected when there was a p-value under 0.00625 for at least one SF-36 domain score, based on the Bonferroni method. All analyses were performed using SAS version 9.1 (SAS Institute Inc., Cary, NC).

## Results

**Figure 1:**
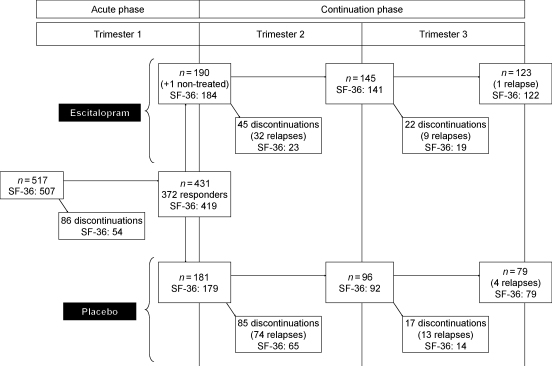
Flow chart summarising number and status of patients participating to the study by treatment arm and trimester

The numbers of patients at different stages of the study are shown in Figure 1. Completion rates for the SF-36 questionnaire were relatively high throughout the study for patients who had not withdrawn. All but 10 patients completed the SF-36 questionnaire at baseline. However, the completion rates were lower among patients who discontinued during the first half of the continuation phase. The questionnaire was completed at the time of discontinuation by 76% of the 169 patients who attended an early discontinuation visit over the whole continuation phase. Among 133 patients who relapsed, 124 (93%) completed the HRQoL assessment.

### Acute phase

**Figure 2:**
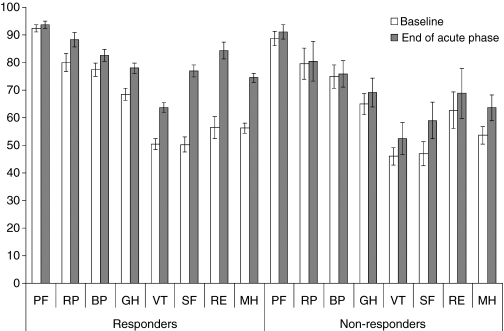
Short form-36 scores at baseline and end of acute phase. PF, Physical Functioning; RP, Role limitations because of Physical problems; BP, Bodily Pain; GH, General perception of Health; SF, Social Functioning; RE, Role limitations because of Emotional problems; MH, Mental Health; VT, Vitality. Numbers of patients at baseline: 366 responders, 141 non-responders; at end of acute phase: 363 responders, 107 non-responders

Short form-36 domain scores at baseline and at the end of acute phase are shown in Figure 2, for responders and for patients who did not respond to treatment or discontinued within 12 weeks (designated as ‘non-responders’ below). For the responders, scores at the end of acute phase were significantly higher than at baseline for all domains except PF (0.00625 significance level). For non-responders, baseline scores were comparable, although slightly lower to those among responders and statistically significant improvements were observed in three domains: GH, SF and MH. No statistically significant decrease in any SF-36 domain score occurred for non-responders. SF-6D utility scores increased from 0.664 to 0.711 among responders and from 0.645 to 0.672 among non-responders. Mean improvements in SF-6D utility adjusted on baseline utility value were 0.049 [95% confidence interval (CI): 0.043–0.055; p < 0.0001] for responders and 0.018 (95% CI: 0.006–0.029; p = 0.0022) for non-responders.

**Figure 3:**
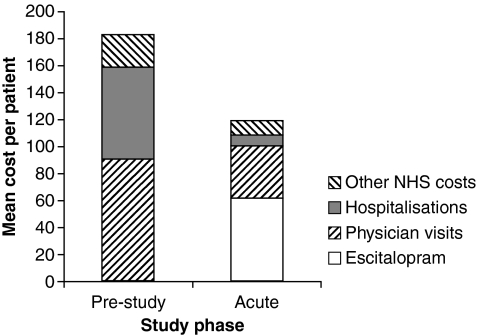
Breakdown of costs (GBP £2006) from a NHS perspective over prestudy and acute phases

Table 2 shows costs over the acute phase compared with those incurred over the 12 weeks preceding the study. Estimated costs were relatively similar from the NHS and the societal perspectives, as healthcare expenditures not included in the NHS perspective accounted for only 5–6% of the societal costs. Nonetheless, the *t*-tests comparing costs between prestudy and acute phases lead to different interpretations according to the perspective. From the societal perspective, estimated costs were significantly lower in the acute phase compared with those in prestudy phase (p = 0.0410). From the NHS perspective, the difference between periods was negative but not statistically significant (p = 0.0587). The analyses in Figure 3 suggest that hospitalisations account for a substantial part of the difference between the two periods. NHS costs were very similar when hospitalisation costs were excluded (mean difference: −£4.36; p = 0.6877).

**Table 2:** Costs (GBP £2006) in responders over prestudy and acute phases

	Prestudy		Acute phase		*t*-test
	*n*	mean (SD)	*n*	mean (SD)	p-value
**Total cost, NHS perspective**					
Including hospitalisations	371	183.32 (640.40)	371	118.82 (183.15)	0.0587
Excluding hospitalisations	371	115.24 (202.70)	371	110.88 (127.27)	0.6877
Total cost, societal perspective	371	196.01 (649.62)	371	125.29 (189.71)	0.0410
Productivity cost	370	273.01 (1385.70)	371	210.58 (1281.39)	0.1440

There was substantial variability in the cost of lost workdays because many patients took no time off work and a few patients were absent over long periods (sometimes, entire trimesters). 68% of patients were in paid employment or self-employed. The mean number of workdays lost decreased from 2.54 over the prestudy phase to 1.96 over the acute phase among responders; this difference was not statistically significant (p = 0.1440).

### Long-term evaluation

Short form-36 scores 24 weeks after randomisation or at last visit, displayed in Table 3, showed that patients who did not relapse reported a better HRQoL than those who did relapse. Significantly higher scores were observed among non-relapsed patients on the four domains more related to MH, with particularly large differences in the social-functioning and role-emotional dimensions. Scores on the physical domains (PF, RP, BP and GH) seemed to be little affected by relapse. The SF-6D utility in non-relapsed patients exceeded the utility in patients who relapsed by 0.026 (p = 0.0007).

**Table 3:** Short form-36 and -6D scores at last assessment

	No relapse (*n* = 227), mean (SD)	Relapse (*n* = 124), mean (SD)	Difference	p-value
PF	94.4 (11.9)	95.0 (9.9)	0.55	0.6443
RP	87.1 (28.4)	85.5 (29.6)	−1.63	0.6123
BP	83.6 (20.4)	79.9 (23.7)	−3.66	0.1306
GH	77.5 (17.3)	74.6 (19.4)	−2.86	0.1584
VT	65.0 (17.3)	58.2 (17.9)	−6.86	0.0005
SF	80.2 (19.9)	64.5 (27.3)	−15.72	< 0.0001
RE	85.8 (27.7)	71.0 (38.5)	−14.79	0.0002
MH	73.8 (16.7)	65.8 (18.6)	−7.98	< 0.0001
SF-6D	0.718 (0.068)	0.691 (0.071)	−0.026	0.0007

PF, Physical Functioning; RP, Role limitations because of Physical problems; BP, Bodily Pain; GH, General perception of Health; SF, Social Functioning; RE, Role limitations because of Emotional problems; MH, Mental Health; VT, Vitality.

Health-related quality of life scores at the last assessment visit were similar to those at randomisation among patients who received long-term treatment with escitalopram (see Table 4). However, for patients randomised to placebo, several mean domain scores decreased significantly from randomisation to the end of the continuation phase or last visit: SF (mean difference: −7.16; p < 0.0001), RE (−7.80; p = 0.0029) and MH (−6.92; p < 0.0001). Differences in SF-36 scores at last visit between treatment groups, adjusted for scores at randomisation, were statistically significant at the 0.00625 level for the SF and MH dimensions, in favour of continuing treatment with escitalopram. In addition, the SF-6D estimate at last assessment was higher by 0.018 in the escitalopram arm (p = 0.0087).

**Table 4:** Short form-36 and -6D scores at randomisation and last visit, by treatment group

	Control		Escitalopram			
	Randomisation (*n* = 179), mean (SD)	Last assessment (*n* = 173), mean (SD)	Randomisation (*n* = 184), mean (SD)	Last assessment (*n* = 178), mean (SD)	Adjusted. difference*	p-value
PF	92.5 (15.1)	93.6 (10.7)	94.7 (9.8)	94.7 (12.3)	−0.55	0.5758
RP	86.6 (27.1)	83.6 (30.5)	89.9 (23.1)	89.5 (25.0)	2.74	0.3603
BP	80.1 (22.2)	80.5 (22.4)	85.1 (18.7)	83.8 (20.5)	−0.77	0.7069
GH	77.4 (17.3)	75.5 (18.9)	78.6 (18.0)	77.2 (17.2)	−1.26	0.3622
VT	63.0 (17.3)	60.4 (17.6)	64.4 (15.9)	63.8 (17.9)	−3.14	0.0640
SF	77.2 (21.1)	70.9 (24.7)	76.6 (20.3)	78.7 (22.5)	−9.70	< 0.0001
RE	84.0 (29.2)	74.2 (35.6)	84.1 (29.3)	84.7 (29.2)	−7.69	0.0167
MH	74.8 (14.8)	68.3 (17.2)	74.2 (16.3)	73.1 (18.1)	−6.48	< 0.0001
SF-6D	0.711 (0.064)	0.698 (0.066)	0.712 (0.064)	0.715 (0.075)	−0.018	0.0087

* Difference in scores at last assessment between treatment groups, adjusted on value at randomisation. PF, Physical Functioning; RP, Role limitations because of Physical problems; BP, Bodily Pain; GH, General perception of Health; SF, Social Functioning; RE, Role limitations because of Emotional problems; MH, Mental Health; VT, Vitality.

For those patients who had not relapsed and completed the SF-36 questionnaire 12 weeks after randomisation (halfway through continuation phase), all mean domain scores were higher in the escitalopram arm, but no difference reached the 0.00625 significance level. The SF domain score was higher by 6.93 in the escitalopram arm (p = 0.0194), the RE domain score by 10.15 (p = 0.0153) and the MH domain score by 6.29 (p = 0.0072); other differences were smaller. The overall difference in SF-6D utility at 12 weeks after randomisation was not statistically significant (mean difference: 0.012; p = 0.1942).

**Figure 4:**
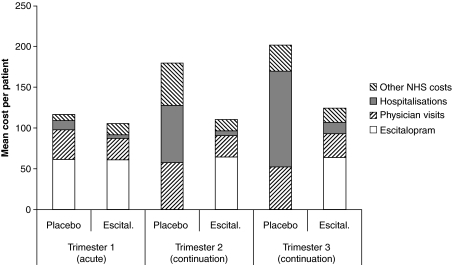
Breakdown of costs (GBP £2006) from NHS perspective over acute and continuation phases, by treatment group

Mean costs among non-relapsed patients were non-significantly lower in the escitalopram group than in the placebo group over the two trimesters of the continuation phase, from the NHS and societal perspectives (see Table 5). As noted for the acute phase, differences between the arms appeared to be largely precipitated by hospitalisation costs (see Figure 4), which occurred in very few patients and introduced substantial uncertainty around estimates of total costs. Similarly, productivity costs were lower in the escitalopram arm over the two trimesters, but the variability in these costs was substantial and differences between treatment arms were not statistically significant.

**Table 5:** Costs (GBP £2006) by treatment group and trimester over continuation phase, in the absence of relapse

	Trimester 2			Trimester 3		
	Placebo (*n* = 96)	Escitalopram (*n* = 145)	p-value	Placebo (*n* = 79)	Escitalopram (*n* = 123)	p-value*
Total cost, NHS perspective, including hospitalisations	180.20	110.53	0.3877	201.74	124.32	0.4431
Total cost, NHS perspective, excluding hospitalisations	110.03	104.73	0.8910	84.50	110.63	0.3511
Total cost, social perspective, including hospitalisations	183.38	115.70	0.4093	206.56	129.67	0.4468
Productivity cost	144.40	115.61	0.8163	186.35	149.40	0.8091

* Student’s *t*-test.

Estimates of productivity costs were lower over each trimester of the randomisation phase than over the open-label phase across the two treatment arms (see Tables 2 and 5). However, the corresponding differences were not statistically significant (p = 0.1454 for trimester 2 vs. open-label and p = 0.6488 for trimester 3 vs. open-label).

## Discussion

This study examined HRQoL and healthcare costs in patients with SAD treated with escitalopram over 12 or 36 weeks. One of the findings of this study was that HRQoL, as measured by the SF-36 instrument, was improved after 12 weeks of treatment with escitalopram. Unsurprisingly, the improvement was greater for treatment responders [i.e. those with a CGI-I of one or two (19)] than non-responders, with differences on SF-36 MH dimensions of between 10 and 30 points, which was greater than the accepted clinically relevant 10-point difference (22). Responders HRQoL subscale scores returned to levels comparable to that of population norms (21). However, the difference between scores at baseline and after acute open-label treatment was statistically significant even for non-responders. Differences in SF and role emotional domain scores were the largest. Furthermore, relapse between 12 and 36 weeks was found to have a negative impact on HRQoL, as shown by the comparison in SF-36 scores between relapsed and non-relapsed patients. Also, the SF-36 dimensions primarily affected by relapse were SF and role emotional. In addition, HRQoL was significantly better in the escitalopram group than in the placebo group at end of continuation phase on two dimensions (SF and MH) and in terms of overall utility, as could be expected given the lower probability of relapse among patients continuing escitalopram beyond 12 weeks. A trend towards higher HRQoL subscale scores and overall utility in the escitalopram group was observed halfway though the continuation phase among non-relapsed patients, suggesting that relapse might not capture the entire effect of escitalopram on HRQoL.

Quantitatively, the results suggested that the effect of escitalopram on HRQoL was somewhat more modest in patients with generalised SAD than in those with generalised anxiety disorder (GAD). A study of escitalopram using a similar design among patients with GAD had shown that the SF-6D score increased from 0.64 at baseline to 0.77 at the end of the open-label period (compared with an increase from 0.66 to 0.71 in responders only in this study). Furthermore, in the GAD study, the decrement in SF-6D utility associated with relapse was 0.06 (compared with 0.03 in SAD).

Total healthcare costs were significantly lower over the acute phase than over the 12 weeks preceding the study from the societal perspective (p = 0.0410). This suggested that the acquisition cost of escitalopram was more than offset by savings in physician-visits and inpatient care. However, the difference in costs between the prestudy and acute phases was not statistically significant from the NHS perspective (p = 0.0587), although costs from the NHS perspective were close to those from the societal perspective. Furthermore, when hospitalisation costs were excluded, healthcare costs were very similar between the two periods. Likewise, productivity costs were not significantly different between the prestudy and acute phases. The comparison of healthcare costs between the acute and continuation phases suggests that costs increased among non-relapsed patients in the placebo arm but not in the escitalopram arm. However, healthcare costs in non-relapsed patients were not significantly different between treatment arms, neither from NHS perspective nor from societal perspective*.* Similarly, a non-significant trend towards lower productivity costs in the escitalopram arm, compared with the placebo arm, was found over the continuation phase.

This study has several limitations, especially concerning the evaluation of the extent to which costs are influenced by response to treatment. The comparison of costs during the acute phase with costs prior to baseline should be interpreted with caution, as study-related visits occurring over the acute phase were not counted, although a proportion of them might have occurred independently of the study. This might account for a part of the difference in societal costs between the prestudy and acute phases, as the difference in costs of physician-visits represented 73% of the difference in societal costs. Thus, if costs of physician-visits were not counted, the total costs in the prestudy and acute phases would be roughly similar, although still slightly lower in the acute phase. Furthermore, the difference in hospitalisation costs, which represented 85% of the difference in total healthcare costs from the societal perspective, is subject to uncertainty. Only 10 patients were hospitalised in the prestudy phase and two in the acute phase (among patients included in the cost analysis). However, the hypothesis that effective treatment is associated with a reduction in hospitalisation costs does seem plausible, as anxiety disorders are known to cause hospitalisations (psychiatric or non-psychiatric) (27). Compared with other anxiety disorders, hospitalisation may be less frequent in SAD as sufferers are understandably frightened by the phobic situations expected in hospital. One study compared use of medical resources between panic disorder and social phobia; hospitalisation rates were similar for both (around 10%) but admission to emergency department was higher for panic disorder patients (21% vs. 7%) (28). It would have been desirable to assess the extent to which costs are influenced by response to treatment by comparing costs in responders and non-responders. However, the available data did not allow for such a comparison. Also, the analysis presented here, comparing costs before and after treatment in responders, relies on the assumption that costs would remain constant from one trimester to the next in the absence of symptomatic improvement.

Costs were estimated by applying unit prices prevailing in the UK for patients from all countries participating in the trial. Among 371 patients who were included in the analysis for the continuation phase, 35 were from the UK. The comparison of costs between treatment groups assumes that there was no interaction between treatment and country on resource utilisation. This assumption has been made in a number of other economic evaluations (29). However, the impact of treatment on resource use may differ among the countries. For example, even if the effect of treatment on the number of physician-visits may be comparable between countries, the effect on the number of psychiatrist consultations is likely to be relatively small in the UK. This could induce bias around cost estimates as psychiatrist visits are more expensive than general practitioner (GP) visits. This problem may have also affected the comparison between prestudy and acute phases. Nevertheless, the cost estimates obtained here are in line with results of a previous study by Patel et al. (8) of the economic consequences of social phobia. He estimated the annual healthcare costs of social phobia at £609 in 1997/1998 prices, which corresponds to approximately £180 per trimester in 2006 prices. This value is very close to our estimate of £183 per trimester for total healthcare costs from the NHS and PSS perspective over the prestudy phase.

It was not possible to distinguish between cases that did not use any resource during a trimester and cases where resource use was not reported. This might have led to an underestimation of costs, irrespective of treatment group or period. However, the extent of this underestimation would have been relatively small if completion rates for the resource utilisation questionnaire were comparable to those for the SF-36 questionnaire. Approximately 95% of patients completed the SF-36 at end of the study or after discontinuation, irrespective of treatment group or presence of relapse. Although approximately one-quarter of those who discontinued did not complete the HRQoL assessment at the time of discontinuing the study treatment, SF-36 data were collected at a later visit in most of these cases.

This study compared costs and HRQoL between treatment groups, but no cost-effectiveness analysis (CEA) was actually performed. The comparison of costs between treatment groups was restricted to non-relapsed patients. Relapses are likely to generate costs as a new therapy will be initiated in many cases and physician-visits will occur at shorter intervals following initiation of new therapy. For example, according to guidelines of the National Institute for Health and Clinical Excellence for the management of GAD in the UK, efficacy and side effects should be reviewed at 2, 4, 6 and 12 weeks following prescription of a new therapy, and subsequently at 8- to 12-week intervals (30). In order to perform a full economic evaluation of escitalopram in the prevention of SAD, it would be necessary to account for the savings realised because of relapses avoided.

Although no CEA was performed in this study, the study provides data that could be useful for future economic evaluations of treatment of SAD, in particular health state utilities. The utility improvement associated with response was estimated at 0.047 (95% CI: 0.040–0.055), by comparison with baseline value, or 0.040 (95% CI: 0.026–0.054) by comparison with the value in non-responders. Furthermore, the utility decrement associated with relapse was −0.026 (95% CI: −0.042 to −0.011). These are probably conservative estimates, as they are based on the SF-6D instrument. The SF-6D utility index has a smaller range than the EQ-5D, another widely used generic HRQoL instrument providing health state utilities. Also, differences in utility between health states based on SF-6D are often smaller than the differences based on the EQ-5D (31,32).

The literature relating to HRQoL in SAD is not very abundant, but some previous studies have shown that SAD is associated with a marked reduction in HRQoL, for example Stein et al. (33), who used the Quality of Well Being Scale and Simon et al. (7), who used the SF-36. Simon et al. reported scores of 59.4 for VT, 65.9 for SF, 67.9 for emotional role and 58.5 for MH, based on 33 patients with SAD. Baseline scores in our sample were below the scores reported by Simon et al., especially the social function score (49.3 on average across responders and non-responders). Thus, our study supports previous evidence indicating that patients with SAD are substantially impaired in their quality of life. The results presented here are consistent with the conclusions of a recent meta-analysis on quality of life in anxiety disorders (34). This meta-analysis demonstrated that the HRQoL impairment of SAD was less multidimensional than that of other anxiety disorders, and more concentrated on the social domain. The quality of life related to physical health was not significantly lower for patients with SAD than for non-clinical controls, whereas a significant difference was found for other disorders. In this study, relapse and treatment were significantly associated with dimensions such as SF and MH, but not with physical dimensions of HRQoL. Unfortunately, this is the only study to our knowledge investigating the impact of treatment on HRQoL, thus no reference is available for comparison.

In conclusion, changes in the severity of SAD translate into changes in overall HRQoL. Patients with SAD responding to acute treatment report improved HRQoL, and those who subsequently relapse have reduced scores. Furthermore, long-term treatment with escitalopram, which is effective in preventing relapse, is associated with a significant benefit in terms of HRQoL. The evidence for treatment-related changes in healthcare costs is limited, but suggests that additional drug acquisition costs may be offset by reductions in costs because of physician-visits and hospitalisation.
